# Gamma counting protocols for the accurate quantification of ^225^Ac and ^213^Bi without the need for a secular equilibrium between parent and gamma-emitting daughter

**DOI:** 10.1186/s41181-022-00174-z

**Published:** 2022-10-23

**Authors:** Dayana Castillo Seoane, Marijke De Saint-Hubert, Stephen Ahenkorah, Clarita Saldarriaga Vargas, Maarten Ooms, Lara Struelens, Michel Koole

**Affiliations:** 1 Unit of Nuclear Medicine and Molecular Imaging, Department of Imaging and Pathology, Katholieke Universiteit Leuven (KUL), Louvain, Belgium; 2 Research Unit in Dosimetric Applications, Belgian Nuclear Research Centre (SCK CEN), Mol, Belgium; 3 NURA Research Group, Belgian Nuclear Research Center (SCK CEN), Mol, Belgium; 4 Unit of Radiopharmaceutical Research, Department of Pharmaceutical and Pharmacological Sciences, Katholieke Universiteit Leuven (KUL), Louvain, Belgium; 5 Department of Medical Imaging, Laboratory for In Vivo Cellular and Molecular Imaging, Vrije Universiteit Brussel (VUB), Brussels, Belgium

**Keywords:** Actinium-225, Bismuth-213, Francium-221, Gamma counter, Radiopharmaceutical quality control, Recoiled daughter effect

## Abstract

**Background:**

Quantification of actinium-225 through gamma counter measurements, when there is no secular equilibrium between actinium-225 and its gamma emitting daughters bismuth-213 and/or francium-221, can provide valuable information regarding the possible relocation of recoiled daughters such that related radiotoxicity effects can be evaluated. This study proposes a multiple time-point protocol using the bismuth-213 photopeak with measurements before secular equilibrium between actinium-225 and bismuth-213, and a single time-point protocol using both the francium-221 and bismuth-213 photopeak while assuming secular equilibrium between actinium-225 and francium-221 but not between bismuth-213 and actinium-225.

**Results:**

Good agreement (i.e. 3% accuracy) was obtained when relying on a multiple time-points measurement of bismuth-213 to quantify both actinium-225 and excess of bismuth-213. Following scatter correction, actinium-225 can be accurately quantified using the francium-221 in a single time-point measurement within 3% of accuracy. The analysis performed on the stability data of [^225^Ac]Ac-DEPA and [^225^Ac]Ac-DOTA complexes, before secular equilibrium between bismuth-213 and actinium-225 was formed, revealed considerable amounts of unbound bismuth-213 (i.e. more than 90%) after 24 h of the radiolabeling most likely due to the recoiled daughter effect.

**Conclusion:**

Both protocols were able to accurately estimate ^225^Ac-activities provided the francium-221 energy window was corrected for the down scatter of the higher-energy gamma-emissions by bismuth-213. This could prove beneficial to study the recoiled daughter effect and redistribution of free bismuth-213 by monitoring the accumulation or clearance of bismuth-213 in different tissues during biodistribution studies or in patient samples during clinical studies. On the other hand, the single gamma counter measurement protocol, although required a 30 min waiting time, is more time and cost efficient and therefore more appropriate for standardized quality control procedures of ^225^Ac-labeled radiopharmaceuticals.

**Supplementary Information:**

The online version contains supplementary material available at 10.1186/s41181-022-00174-z.

## Introduction

Targeted alpha-therapy (TAT) has shown promising results when overcoming resistance to β-emitters in clinical applications (Kratochwil et al. 2014; Ballal et al. 2020). The efficiency of α-particles relies on their short penetration range within tissue (40–100 μm, Allen et al. 2014) and their high linear energy transfer (LET). ^225^Ac (actinium-225) is considered a promising candidate for TAT and a highly cytotoxic radionuclide due to its relatively long half-life (9.9 days) and the yield of a total of four α particles ^221^Fr (francium-221): 4.9 min half-life, 6 MeV α-particle; ^217^At (astatine-217): 32.3 ms half-life, 7 MeV α-particle; ^213^Bi (bismuth-213): 45.6 min half-life, 6 MeV α-particle; ^213^Po (polonium-213): 4.2 µs half-life, 8 MeV α-particle) in its decay chain. In addition, two gamma rays are emitted in the decay chain, 218 keV [11.4%] by ^221^Fr and 440 keV [25.9%] by ^213^Bi, which can be used for activity measurements. Indeed, most preclinical studies report ^225^Ac activities which are based on activity measurements of ^213^Bi or ^221^Fr while assuming secular equilibrium (SEq) between ^225^Ac and the measured daughter (Kruijff et al. 2019; Miederer et al. 2004a; Borchardt et al. 2003; Beyer et al. 1990), which means activity measurements based on the gamma emissions by either ^221^Fr or ^213^Bi are approximately equal to the activity of ^225^Ac. However, emission of a high-energy α particle can cause nuclear recoil effect (Kozempel et al. 2018). This recoil energy experienced by the daughter nuclei is sufficient to break the chemical bond between the daughter and the targeting vector (Kruijff et al. 2015), resulting in the so-called recoiled daughter effect (RDE) and causing at least partial release of radioactive daughter nuclei from the original targeting molecule or delivery vehicle (Kruijff et al. 2015). Loss of affinity to the molecular carrier can lead to a redistribution of recoiling radioactive daughters and induce radiation related side effects, such that RDE is often assumed to be the main cause of radiotoxicity for the organs at risk (OAR) and one of the main reasons for limiting the amount of activity of ^225^Ac-labeled radionuclides administered to patients (Ballal et al. 2020; Kratochwil et al. 2017, 2015; Khreish et al. 2020; Cordier et al. 2010). Redistribution of recoiled daughter radionuclides can also offset the SEq between ^225^Ac and daughter radionuclides, such that indirect measurement of the activity concentration of ^225^Ac by measuring the activity concentration of its gamma-ray emitting daughters ^221^Fr or ^213^Bi can be biased. Only a few studies (Kruijff et al. 2019; Miederer et al. 2004a, 2008; Poty et al. 2019; Nedrow et al. 2017; Schwartz et al. 2011; Song et al. 2009) have considered measurements before and during SEq between ^225^Ac and ^213^Bi, to evaluate the relocation of ^213^Bi, and in very limited cases also between ^225^Ac and ^221^Fr, to evaluate the relocation of ^221^Fr. Recoiling ^213^Bi was reported to have affinity to kidney tissue (Kruijff et al. 2019; Song et al. 2009; Drecoll et al. 2009), while ^221^Fr was associated with uptake in the gastrointestinal tract (Miederer et al. 2004b) and kidneys (Song et al. 2009). However, it should be noted that relocation of ^221^Fr is generally not considered as relevant because of the very short physical half-life of ^221^Fr (4.9 min), such that ^221^Fr is considered as the closest proxy to quantify ^225^Ac activity concentrations.

In the context of TAT, gamma counting (GC) is a frequently used technique for ex vivo quantification of ^225^Ac-labeled radiopharmaceuticals (Castillo Seoane et al. 2020), especially since in vivo nuclear imaging techniques, such as single-photon emission computed tomography (SPECT), have limited potential to allow accurate quantitative imaging of ^225^Ac concentrations in tissues because of the low branching ratio of gamma-emissions in the decay chain and the low administered activities. Therefore, other measurement techniques, such as GC, represent an asset for dosimetry and radiotoxicity estimates, both preclinically and clinically. In a preclinical setting, GC allows biodistribution studies of ^225^Ac-labeled radiopharmaceuticals to estimate the absorbed doses by tumoral and healthy tissue. In addition, GC provides valuable information regarding the possible relocation of recoiled daughters, which is of interest to evaluate related radiotoxicity effects. In a clinical setting, GC measurements of blood and urine samples can be considered to determine plasma and renal clearance of radiopharmaceuticals, which in turn can be used for compartmental modeling of pharmacokinetics to estimate the radiation burden to OAR (Siegel et al. 1999).

In addition, GC measurements play an important role in the quality control (QC) of ^225^Ac-labeled radiopharmaceuticals to confirm sufficiently high radiochemical yield (RCY) before administration to patients. For this purpose, the activity distribution on an instant thin-layer liquid chromatography (iTLC) strip is analyzed using GC measurements to estimate the fraction of bound and unbound ^225^Ac and its daughter ^213^Bi. These GC measurements are usually based on the gamma emissions by ^221^Fr, which is at SEq with ^225^Ac within approximately six half-lives of ^221^Fr (~ 30 min) (Hooijman et al. 2021).

However, limited research has been done on GC measurements to quantify ^225^Ac activity when there is no SEq between ^225^Ac and its gamma emitting daughters ^213^Bi and/or ^221^Fr. Generally, GC measurements are delayed ensuring sufficient time for ^213^Bi to reach SEq with ^225^Ac, and to provide an unbiased estimation of the ^225^Ac-activity. The limitation of this approach is that, once in SEq, any additional ^213^Bi activity which was originally present in the measured sample before the start of the GC measurements cannot be traced back. Nonetheless, there is a growing interest to quantify ^213^Bi activity which is not related to the parent–daughter decay of ^225^Ac, but that is generated by the potential RDE and relocation of ^213^Bi. Therefore, the aim of this study is to develop and validate GC protocols to accurately quantify both ^225^Ac activity and a potential accumulation or clearance of ^213^Bi activity compared to SEq between ^225^Ac and ^213^Bi. We proposed (1) a multiple time-point protocol using the ^213^Bi photopeak with measurements before SEq between ^225^Ac and ^213^Bi, and (2) a single time-point protocol using both the ^221^Fr and ^213^Bi photopeaks, while assuming SEq between ^225^Ac and ^221^Fr but not between ^213^Bi and ^225^Ac. Using these two protocols, we evaluated the amount of unbound ^213^Bi for two different chelators [^225^Ac]Ac-DEPA(7-[2-(bis-carboxymethyl-amino)-ethyl]-4,10-bis-carboxymethyl-1,4,7,10- tetraazacyclododec-1-yl-acetic acid) and [^225^Ac]Ac-DOTA (1,4,7, 10-Tetraazacyclododecane-1, 4,7,10-tetracetic acid) using iTLC strips after incubation in human serum to evaluate stability and RDE as part of QC tests after radiolabeling.

## Materials and methods

### Radioisotopes and preparation of [^225^Ac]-labeled constructs

^225^Ac samples were obtained from recurrent (trimestral, 6 MBq) milkings of an in-house ^229^Th stock obtained from a processed ^229^Th capsule as described by Boden et al. (2017). Milkings were performed with the aid of a tandem system of extraction chromatography columns using TEVA and DGA columns obtained from TrisKem, France. A first batch of ^225^Ac was used to evaluate the GC linearity and the effect of the sample volume variation on the GC detection efficiency (for details on the sample volume variations see Additional file 1).

Additionally, ± 1.3 MBq of ^225^Ac was used to elute 1.19 MBq of ^213^Bi (using 0.1 M HCl/ 0.1 M NaI) from an ^225^Ac/^213^Bi generator loaded with an AG MP-50 cation exchange resin (Ahenkorah et al. 2021). The eluate ^213^BiI_4_^−^/^213^BiI_5_^2−^ was used to prepare two samples, one containing ^225^Ac in SEq with ^213^Bi and ^221^Fr for the GC calibration, and one containing pure ^213^Bi to determine the photon scatter contribution of ^213^Bi gamma-emissions in the photopeak window of ^221^Fr.

Two other solutions of ^225^Ac doped with additional ^213^Bi were prepared to create a mixture of ^225^Ac in SEq with ^213^Bi and additional, pure ^213^Bi, to simulate non-equilibrium conditions between ^225^Ac and ^213^Bi. As such, the total activity of ^213^Bi was given by:

1 $$A_{{{\text{total}}}} = A_{{21{\text{3}}_{{{\text{Bi}}}} }} + A_{{225_{{{\text{Ac}}}} }}$$

where $A_{{225_{{{\text{Ac}}}} }}$ is the activity of ^213^Bi in SEq with ^225^Ac. This was done by starting from a solution (~ 0.5 mL) of ^225^Ac in SEq with its decay progeny and adding 0.2 mL (and 0.3 mL) of the stock solution of pure ^213^Bi to 0.3 mL (and 0.2 mL) of the solution of ^225^Ac to obtain two samples with additional ^213^Bi activity (total sample volume ~ 0.5 mL).

^225^Ac-constructs were synthesized by adding [^225^Ac]Ac(NO_3_)_3_ (1–2 MBq, 100 μL, 0.05 M) to a Tris–HCl buffer (0.37 M, pH 8.5, chelex treated) solution containing DEPA or DOTA (50 µM) and reacting in a glass vial for 60 min at 95 °C. ^225^Ac-constructs were purified using a C_18_ Plus Sep-Pak cartridge (Waters Co., Milford, MA, USA) by loading the reaction mixture, rinsing with water (5 mL) to remove unreacted [^225^Ac]Ac(NO3)_3_, and eluting the purified complex with absolute ethanol (0.5 mL) as described by Cassells et al. (2021). After purification, radiolabeled aliquots were applied immediately to the test media.

The radioactive composition of all reference samples (i.e. stock solutions) were determined via high-resolution gamma spectrometry analysis using a high-purity germanium (HPGe) detector (Mirion-Canberra, Meriden, USA) calibrated for photon energy and detection efficiency. ^225^Ac quantities were determined indirectly via measurements of ^221^Fr and ^213^Bi photopeaks once SEq was established. ^213^Bi quantities were determined via measurement of a pure ^213^Bi sample as fast as possible after elution. The content mass of the sample was verified gravimetrically and the exact volumes of the radioactive samples in each test tube were determined by weighing each tube before and after filling with an analytical balance (model ABP-200-4M; KERN & SOHN GmbH, Germany). The reference activity in each test tube was determined from the volume of each sample and the activity concentration of the reference stock solution. The relative statistical uncertainty of the reference activity concentration of each of the calibration stock solutions was always within 3.2% at 95% CI (coverage factor *k* = 2).

### Gamma counter measurements

Gamma counter activity measurements were done in a 2480 Wizard^2^ gamma counter (Perkin Elmer, Waltham, MA, USA) using a measurement protocol optimized to limit the overall measurement uncertainty (cfr. details in the Additional file 1). This gamma counter consists of a single 75-mm-diameter and 80-mm-high NaI(Tl) well-type detector. Each radioactive sample was measured for 60 s in a standard tube (5 mL plastic vials of 75 mm height and 12 mm diameter), using a fixed energy window of 175–282 keV (${\text{EW}}_{{221_{{{\text{Fr}}}} }}$) and 378–520 keV (${\text{EW}}_{{213_{{{\text{Bi}}}} }}$) corresponding to the main photopeaks of ^221^Fr (218 keV) and ^213^Bi (440 keV), respectively. The measurement in each EW setting was automatically corrected for dead time, background, and measurement time and expressed as counts per minute (CPM).

First, the linear range of GC measurements for the two energy windows was determined, together with the GC calibration factor (CF). For the impact of different sample volumes on the detection efficiency, we refer to the Additional file 1.

Assuming SEq between ^225^Ac and ^213^Bi (and thus ^221^Fr), the calibration factors for ${\text{EW}}_{{213_{{{\text{Bi}}}} }}$ ($CF_{{213_{{{\text{Bi}}}} }}$) and ${\text{EW}}_{{221_{{{\text{Fr}}}} }}$ ($CF_{{221_{{{\text{Fr}}}} }}$) were determined from GC measurements as:

2 $$CF_{{213_{{{\text{Bi}}}} }} {\text{ }} = {\text{ }}\frac{{CPM_{{{\text{EW}}_{{213_{{{\text{Bi}}}} }} }} }}{{A_{{225_{{{\text{Ac}}}} }} }}$$

3 $$CF_{{221_{{{\text{Fr}}}} }} {\text{ }} = {\text{ }}\frac{{CPM_{{{\text{EW}}_{{221_{{{\text{Fr}}}} }} }} }}{{A_{{225_{{{\text{Ac}}}} }} }}$$

**Fig. 1:**
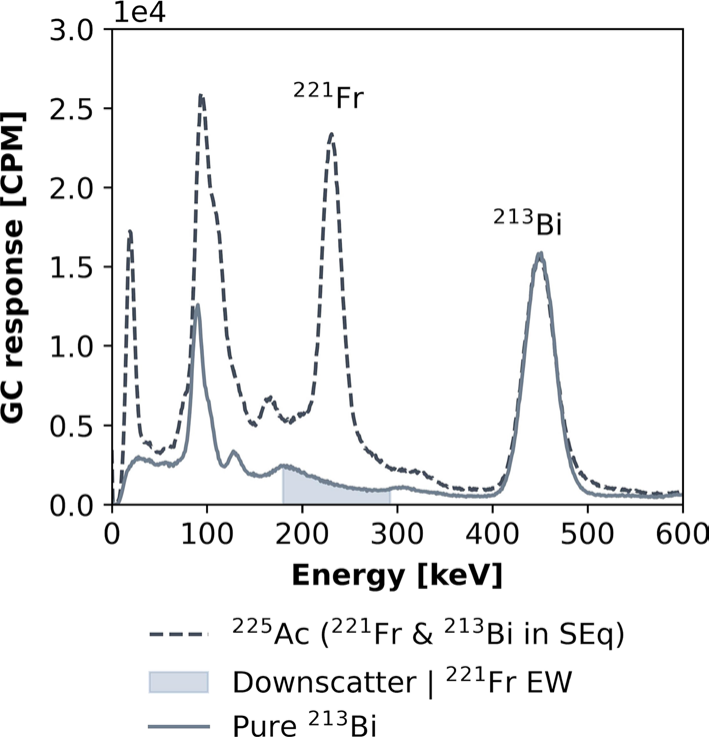
GC photon energy spectrum measured for ^225^Ac, in SEq with both ^221^Fr and ^213^Bi, and for pure ^213^Bi

If there is SEq between ^225^Ac and ^221^Fr but not between ^225^Ac and ^213^Bi, we want to have the CPM measured with ${\text{EW}}_{{221_{{{\text{Fr}}}} }}$ to be independent of the ^213^Bi activity. Therefore, we needed to correct for the down scatter of the higher high-energy gamma rays of ^213^Bi into the ${\text{EW}}_{{221_{{{\text{Fr}}}} }}$ (Fig. 1). Hence, the scatter contribution of ^213^Bi to ${\text{EW}}_{{221_{{{\text{Fr}}}} }}$ ($CPM_{{213_{{{\text{Bi}}}} \to {\text{EW}}_{{221_{{{\text{Fr}}}} }} }}$) was determined as function of $CPM_{{{\text{EW}}_{{213_{{{\text{Bi}}}} }} }}$ by performing GC measurements using both ${\text{EW}}_{{221_{{{\text{Fr}}}} }}$ and ${\text{EW}}_{{213_{{{\text{Bi}}}} }}$ for a pure ^213^Bi sample which was measured for more than 5 h to cover different $CPM_{{{\text{EW}}_{{213_{{{\text{Bi}}}} }} }}$ values. As such GC measurements using the ${\text{EW}}_{{221_{{{\text{Fr}}}} }}$ can be corrected for the ^213^Bi down scatter by subtracting the activity-dependent scatter contribution of ^213^Bi to the ${\text{EW}}_{{221_{{{\text{Fr}}}} }}$ using the following expression:

4 $$CPM_{{{\text{SC,EW}}_{{221_{{{\text{Fr}}}} }} }} = CPM_{{{\text{EW}}_{{221_{{{\text{Fr}}}} }} }} - CPM_{{213_{{{\text{Bi}}}} \to {\text{EW}}_{{221_{{{\text{Fr}}}} }} }}$$

This resulted in a calibration factor for the scatter-corrected ${\text{EW}}_{{221_{{{\text{Fr}}}} }}$ to accurately estimate ^225^Ac activities independent of ^213^Bi activities:

5 $$CF_{{{\text{SC,}}221_{\text{Fr}}}} = \frac{{CPM_{{{\text{SC,EW}}_{{221_{{{\text{Fr}}}} }} }} }}{{A_{{225_{{{\text{Ac}}}} }} }}$$

## Multiple time-point GC measurements to quantify ^225^Ac doped with ^213^Bi

Immediately after preparation of the two samples with an additional amount of ^213^Bi activity (compared to ^213^Bi activity in SEq with ^225^Ac), GC measurements were performed sequentially, at intervals of 5 min, for a total duration of 5–6 h after preparation, using the ${\text{EW}}_{{213_{{{\text{Bi}}}} }}$ and corresponding $CF_{{213_{{{\text{Bi}}}} }}$*.* Given $A_{{225_{{{\text{Ac}}}} }}^{0}$, the initial activity of ^213^Bi in SEq with ^225^Ac, and $A_{{213_{{{\text{Bi}}}} }}^{0}$, the additional amount of ^213^Bi activity at the start of the measurement (*t* = 0), the total activity of ^213^Bi at time *t* is given by (see Eqs. 1, 2):

6 $$\frac{{CPM_{{{\text{EW}}_{{213_{{{\text{Bi}}}} }} }} (t)}}{{CF_{{213_{{{\text{Bi}}}} }} }} = A_{{{\text{total}},213_{{{\text{Bi}}}} }} \left( t \right) = A_{{225_{{{\text{Ac}}}} }}^{0} \times {\text{e}}^{{ - \lambda _{{225_{{{\text{Ac}}}} }} t}} + A_{{213_{{{\text{Bi}}}} }}^{0} \times {\text{e}}^{{ - \lambda _{{213_{{{\text{Bi}}}} }} t}}$$

with $CPM_{{{\text{EW}}_{{213_{{{\text{Bi}}}} }} }} {\text{(}}t{\text{)}}$ the GC measurement at time *t* using ${\text{EW}}_{{213_{{{\text{Bi}}}} }}$ and with $\lambda _{{225_{{{\text{Ac}}}} }}$ and $\lambda _{{213_{{{\text{Bi}}}} }}$ the physical decay constants for ^225^Ac and ^213^Bi respectively. Therefore, GC measurements using ${\text{EW}}_{{213_{{{\text{Bi}}}} }}$ were analyzed as a function of time *t* after start of the measurement and used to determine the initial ^213^Bi activity in SEq with ^225^Ac and the additional amount of ^213^Bi at the start of the GC measurement. This was done by non-linear least squares fitting (GraphPad Prism version 9.1.0) of a double exponential function to the GC measurements at different time-points with the physical decay constants fixed.

### Single time-point GC measurements to quantify ^225^Ac doped with ^213^Bi

In the second approach, a single time-point GC measurement using both ${\text{EW}}_{{221_{{{\text{Fr}}}} }}$ and ${\text{EW}}_{{213_{{{\text{Bi}}}} }}$ was considered, which theoretically should allow an estimation of both ^225^Ac activity in SEq with ^213^Bi and an additional amount of ^213^Bi activity. This approach assumes one single GC measurement after SEq is reached between ^225^Ac and ^221^Fr (after ~ 30 min), but as soon as possible thereafter, to ensure that any ^213^Bi present in the sample has not yet reached SEq with ^225^Ac. This way, one can still differentiate between the amount of ^213^Bi activity in SEq with ^225^Ac and the additional amount of ^213^Bi activity. If we assume the single time-point GC measurement at time *t* after sample preparation, the additional amount of ^213^Bi activity at the start of the measurement ($A_{{213_{{{\text{Bi}}}} }}^{0}$) can be estimated using the ${\text{EW}}_{{213_{{{\text{Bi}}}} }}$ once the initial activity of ^225^Ac ($A_{{225_{{{\text{Ac}}}} }}^{0}$) at the start of the measurement is known:

7 $$A_{{213_{{{\text{Bi}}}} }}^{0} {\text{ }} = {\text{ }}\left( {\frac{{CPM_{{{\text{ EW}}_{{213_{{{\text{Bi}}}} }} }} {\text{(}}t{\text{)}}}}{{CF{\text{ }}_{{213_{{{\text{Bi}}}} }} }} - {\text{ }}A_{{225_{{{\text{Ac}}}} }}^{0} \times {\text{e}}^{{ - \lambda _{{225_{{{\text{Ac}}}} }} t}} } \right) \times {\text{e}}^{{\lambda _{{213_{{{\text{Bi}}}} }} t}}$$

with $CPM_{{{\text{EW}}_{{213_{{{\text{Bi}}}} }} }} {\text{(}}t{\text{)}}$ the GC measurement at time *t* using ${\text{EW}}_{{213_{{{\text{Bi}}}} }}$ and with $\lambda _{{225_{{{\text{Ac}}}} }}$ and $\lambda _{{213_{{{\text{Bi}}}} }}$ the physical decay constant for ^225^Ac and ^213^Bi respectively. In turn, the initial activity of ^225^Ac ($A_{{225_{{{\text{Ac}}}} }}^{0}$) can be estimated using the ${\text{EW}}_{{221_{{{\text{Fr}}}} }}$ either from:

8 $$A_{{225_{{{\text{Ac}}}} }}^{0} = \frac{{CPM_{{{\text{ EW}}_{{221_{{{\text{Fr}}}} }} }} (t)}}{{CF_{{221_{{{\text{Fr}}}} }} }} \times {\text{e}}^{{\lambda _{{225_{{{\text{Ac}}}} }} t}}$$

with $CPM_{{{\text{EW}}_{{221_{{{\text{Fr}}}} }} }} {\text{(}}t{\text{)}}$ the GC measurement at time *t* using ${\text{EW}}_{{221_{{{\text{Fr}}}} }}$ without scatter correction (see Eq. 3), or from:

9 $$A_{{225_{{{\text{Ac}}}} }}^{0} = \frac{{CPM{\text{ }}_{{{\text{SC,EW}}_{{221_{{{\text{Fr}}}} }} }} (t)}}{{CF_{{{\text{ SC,221}}_{{{\text{Fr}}}} }} }} \times {\text{e}}^{{\lambda _{{{\text{ 225}}_{{{\text{Ac}}}} {\text{ }}}} t}}$$

with $CPM_{{{\text{SC,EW}}_{{221_{{{\text{Fr}}}} }} }} (t)$ the GC measurement at time *t* using ${\text{EW}}_{{221_{{{\text{Fr}}}} }}$ with scatter correction (see Eq. 5). In both Eq. 8 and Eq. 9, $\lambda _{{225_{{{\text{Ac}}}} }}$ represents the physical decay constant for ^225^Ac.

Both Eqs. 8 and 9 were considered to estimate the initial activity of ^225^Ac using ${\text{EW}}_{{221_{{{\text{Fr}}}} }}$.

### Quantification of the bound fraction of ^225^Ac and ^213^Bi during radiopharmaceutical QC of [^225^Ac]Ac-DEPA and [^225^Ac]Ac-DOTA complexes

**Fig. 2:**
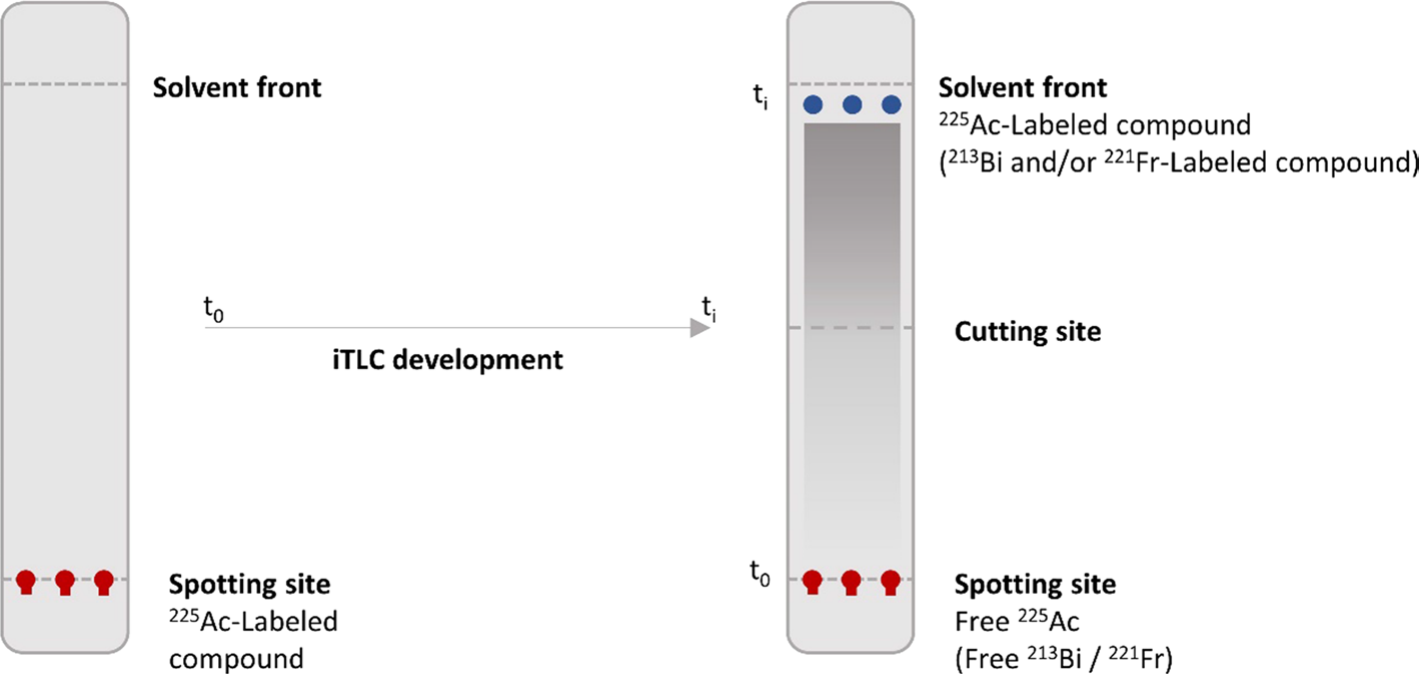
Schematic representation of the migration of radiolabeled compounds in the iTLC strip. Upon exposure of the strip to the mobile phase, unbound radionuclides will remain in the spotting site, whereas bound radionuclides will move with the solvent front to the upper part of the iTLC strip

As a direct application, we performed GC measurements at multiple time-points during the stability test of two constructs, [^225^Ac]Ac-DEPA and [^225^Ac]Ac-DOTA, as part of radiopharmaceutical QC. For this stability test, two ethanolic solutions of 50 μL, each containing one of the constructs, were added immediately after purification to 450 μL of human serum and incubated at 37 °C. Samples were collected at 15 min and 24 h post reaction. The RCY of each reaction mixture was determined by instant thin-layer liquid chromatography (iTLC-SG, Varian, Diegem, Belgium) with an elution chamber using acetonitrile/water (75%/25% v/v) such that bound ^225^Ac and bound daughter radionuclides will migrate with the solvent front to the upper part of the iTLC strip, while unbound radionuclides will remain at the lower part where the mixture was originally spotted (Fig. 2) (Cassells et al. 2021). Once the solvent front reached the 1-cm mark of the iTLC strip at time *t*_i_ (cfr solvent front Fig. 2), the iTLC strip is removed from the mobile phase and cut in half. Immediately after, the activity of the upper and lower parts of the iTLC strip were measured with GC using both ${\text{EW}}_{{221_{{{\text{Fr}}}} }}$ and ${\text{EW}}_{{213_{{{\text{Bi}}}} }}$ with the previously described energy window settings.

The ^225^Ac activity was estimated for each part of the iTLC strip using a single time-point GC measurement after more than 5 h to ensure SEq between ^225^Ac and ^213^Bi such that the ^225^Ac activity could be estimated from the CPM data acquired with ${\text{EW}}_{{213_{{{\text{Bi}}}} }}$ and extrapolated to the start of the iTLC elution at *t*_0_.

In addition, a single time-point measurement was performed at 30 min after starting the iTLC elution to ensure SEq between ^225^Ac and ^221^Fr such that the ^225^Ac activity could be estimated from the CPM data acquired with ${\text{EW}}_{{221_{{{\text{Fr}}}} }}$ with (Eq. 9) and without scatter correction (Eq. 8). Next, the increase (or decrease) in ^213^Bi activities compared to SEq (dependent on the part of the strip) was estimated from the CPM acquired with ${\text{EW}}_{{213_{{{\text{Bi}}}} }}$. In case of an increase of ^213^Bi, the ^213^Bi activity at the start of the iTLC elution (*t*_0_) was estimated using Eq. 7.

For the upper part of the iTLC strips obtained after 24 h of incubation in human serum, multiple time-point measurements were performed before SEq between ^225^Ac and ^213^Bi (> 5 h) while only the CPM data acquired with the ${\text{EW}}_{{213_{{{\text{Bi}}}} }}$ were used for the analysis. In case of increased or reduced ^213^Bi activity compared to SEq with ^225^Ac, fitting of a double exponential function (i.e. Eq. 6) was performed by non-linear least squares fitting (GraphPad Prism version 9.1.0) to the multiple GC measurements at different time-points. For both ^225^Ac and ^213^Bi, the bound fraction was determined as the ratio of the activity of either radionuclide measured for the upper part of the iTLC over the sum of activities measured for both the upper and the lower part of the iTLC.

## Results

### ^225^Ac/^213^Bi samples and [^225^Ac]-labeled constructs

Table 1 gives an overview of the different samples for GC measurements and the activity at the start of the measurements. To evaluate the GC linearity a total of 19 test samples were prepared from sample 1. The samples were prepared with equal volume (0.5 mL), to avoid a volume effect on sensitivity, and with varying activities ranging from 1 Bq up to 500 kBq of ^225^Ac in SEq with ^221^Fr and ^213^Bi.

**Table 1:** Overview of the different samples for GC measurements and the activity at the start of the measurements

Sample	^225^Ac (in SEq with ^213^Bi)	Added ^213^Bi	Experiment
1	1.5 MBq (total)	0 kBq	GC calibration
2	0 kBq	117 kBq	Quantifying down scatter of higher-energy gamma emissions by ^213^Bi in ${\text{EW}}_{{221_{{{\text{Fr}}}} }}$
3	94 kBq	55 kBq	Simulating non-SEq conditions between ^225^Ac and ^213^Bi
4	116 kBq	32 kBq	
5	1–2 MBq	0 kBq	Radiolabeling of [^225^Ac]Ac-DOTA and incubation in human serum for 15 min and 24 h
6	1–2 MBq	0 kBq	Radiolabeling of [^225^Ac]Ac- DEPA and incubation in human serum for 15 min and 24 h

Specifications of the samples used for determining the linear range of GC measurements and evaluating the impact of different sample volumes on GC measurements is given in the Additional file 1

### Gamma counter measurements

**Fig. 3:**
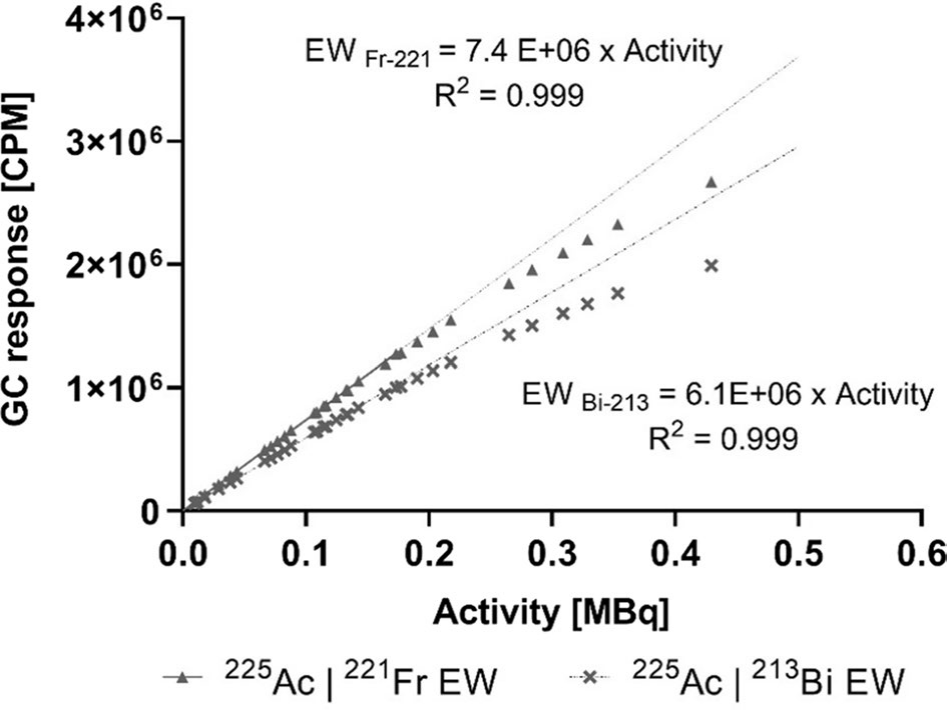
GC response for ^225^Ac in SEq with ^221^Fr and ^213^Bi, measured with both EWs of ^221^Fr and ^213^Bi. Error bars represent the standard deviation provided by the GC software

**Fig. 4:**
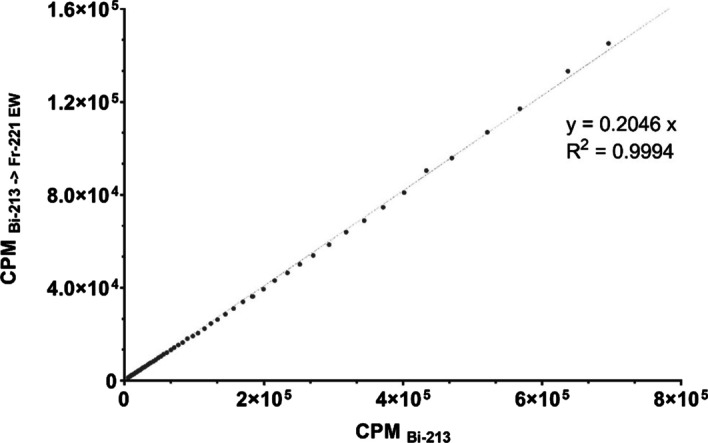
Count rate of the down scatter of gamma emissions by ^213^Bi in the ${\text{EW}}_{{221_{{{\text{Fr}}}} }}$ ($CPM_{{213_{{{\text{Bi}}}} \to {\text{EW}}_{{221_{{{\text{Fr}}}} }} }}$) for different count rates of ^213^Bi measured with the ${\text{EW}}_{{213_{{{\text{Bi}}}} }}$ ($CPM_{{{\text{ 213}}_{{{\text{Bi}}}} }}$)

For both EW setting for ^221^Fr and ^213^Bi, the GC response expressed as count rate (CPM) was measured as a function of the different activities obtained from sample 1 which was cross-calibrated with a standard HPGe detector (see Fig. 3). A non-linearity of more than 3% in the GC response was observed for activities above 150 kBq, resulting in a CPM underestimation of up to 17% and 25% for activities around 0.5 MBq when using the ${\text{EW}}_{{221_{{{\text{Fr}}}} }}$ and the ${\text{EW}}_{{213_{{{\text{Bi}}}} }}$ respectively. Therefore, GC response was considered linear up to 150 kBq ^221^Fr and ^213^Bi, in SEq with ^225^Ac. For this linear range, CFs were determined by linear regression, resulting in GC CFs for the EWs of ^213^Bi ($CF_{{213_{{{\text{Bi}}}} }}$) and ^221^Fr ($CF_{{221_{{{\text{Fr}}}} }}$) of 6.1E+06 CPM/MBq and 7.4E+06 CPM/MBq, respectively. The CPMs obtained with GC using the ${\text{EW}}_{{221_{{{\text{Fr}}}} }}$ ($CPM_{{{\text{ 213}}_{{{\text{Bi}}}} \to {\text{EW}}_{{221_{{{\text{Fr}}}} }} }}$) are shown as a function of different CPM measured with ${\text{EW}}_{{213_{{{\text{Bi}}}} }}$ ($CPM_{{213_{{{\text{Bi}}}} }}$) for different ^213^Bi activities obtained from a cross-calibration with the HPGe detector (see Fig. 4). Results indicated a clear, linear relationship between $CPM_{{213_{{{\text{Bi}}}} \to {\text{EW}}_{{221_{{{\text{Fr}}}} }} }}$ and $CPM_{{213_{{{\text{Bi}}}} }}$ (linear regression, slope = 0.205 ± 0.001, R^2^ > 0.99) which can be used to estimate the scatter contribution of ^213^Bi to GC measurements in the ${\text{EW}}_{{221_{{{\text{Fr}}}} }}$. This scatter fraction can be subtracted from ${\text{EW}}_{{221_{{{\text{Fr}}}} }}$, and ^225^Ac activities can be accurately estimated even when there is no SEq between ^225^Ac and ^213^Bi (Eq. 9). The corresponding GC calibration factor for the ${\text{EW}}_{{221_{{{\text{Fr}}}} }}$, when a correction for ^213^Bi photon down scatter is applied, was determined as 6.1E+06 CPM/MBq.

### Multiple time-point GC measurements to quantify ^225^Ac doped with ^213^Bi

**Fig. 5:**
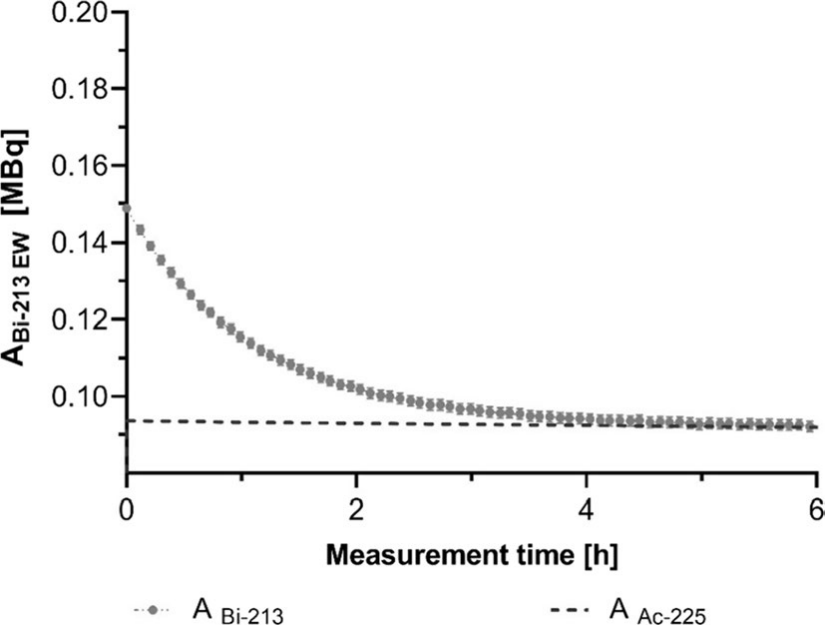
^213^Bi activities of sample 3 (see Table 1) containing ^213^Bi in SEq with ^225^Ac and an added amount of ^213^Bi. GC measurements were performed at different time-points before and after SEq between ^213^Bi and ^225^Ac using the ${\text{EW}}_{{213_{{{\text{Bi}}}} }}$. Error bars represent the standard deviation provided by the GC software

For the multiple time-point approach, activities measured by the gamma counter using ${\text{EW}}_{{213_{{{\text{Bi}}}} }}$ are plotted as a function of the measurement time for the sample 3 (see Table 1) containing ^213^Bi activity in SEq with ^225^Ac and an additional amount of ^213^Bi activity (see Fig. 5). Based on a bi-exponential fit with fixed physical decay constants for ^225^Ac and ^213^Bi (cfr Eq. 6), ^225^Ac and ^213^Bi activities were estimated (see Table 2), both with a relative percentage difference lower than 3% compared to the reference activities determined with the HPGe detector.

**Table 2:** Multiple and single time-point (at 30 min) estimation of the ^225^Ac and ^213^Bi activity for sample 3 and 4 containing ^213^Bi in SEq with ^225^Ac and an additional amount of ^213^Bi

	CPM	^213^Bi (SEq with ^225^Ac)		Added ^213^Bi	
		kBq	%diff	kBq	%diff
Sample 3					
Multiple time-points	$CPM_{{{\text{EW}}_{{213_{{{\text{Bi}}}} }} }}$	93.7	< 3	54.9	< 3
Single time-point at 30 min	$CPM_{{{\text{EW}}_{{213_{{{\text{Bi}}}} }} }}$, $CPM_{{{\text{EW}}_{{221_{{{\text{Fr}}}} }} }}$	101.8	9	47.0	− 15
Single time-point at 30 min (scatter corrected)	$CPM_{{{\text{EW}}_{{213_{{{\text{Bi}}}} }} }}$, $CPM_{{{\text{SC,EW}}_{{221_{{{\text{Fr}}}} }} }}$	93.4	< 3	55.3	< 3
Sample 4					
Multiple time-points	$CPM_{{{\text{EW}}_{{213_{{{\text{Bi}}}} }} }}$	116.2	< 3	32.9	< 3
Single time-point at 30 min	$CPM_{{{\text{EW}}_{{213_{{{\text{Bi}}}} }} }}$, $CPM_{{{\text{EW}}_{{221_{{{\text{Fr}}}} }} }}$	122.9	6	28.4	− 11
Single time-point at 30 min (scatter corrected)	$CPM_{{{\text{EW}}_{{213_{{{\text{Bi}}}} }} }}$, $CPM_{{{\text{SC,EW}}_{{221_{{{\text{Fr}}}} }} }}$	116.7	< 3	32.8	< 3

Corresponding CPM approaches are indicated and the relative difference (%diff) compared to the reference activities in both samples

### Single time-point GC measurements to quantify ^225^Ac doped with ^213^Bi

**Fig. 6:**
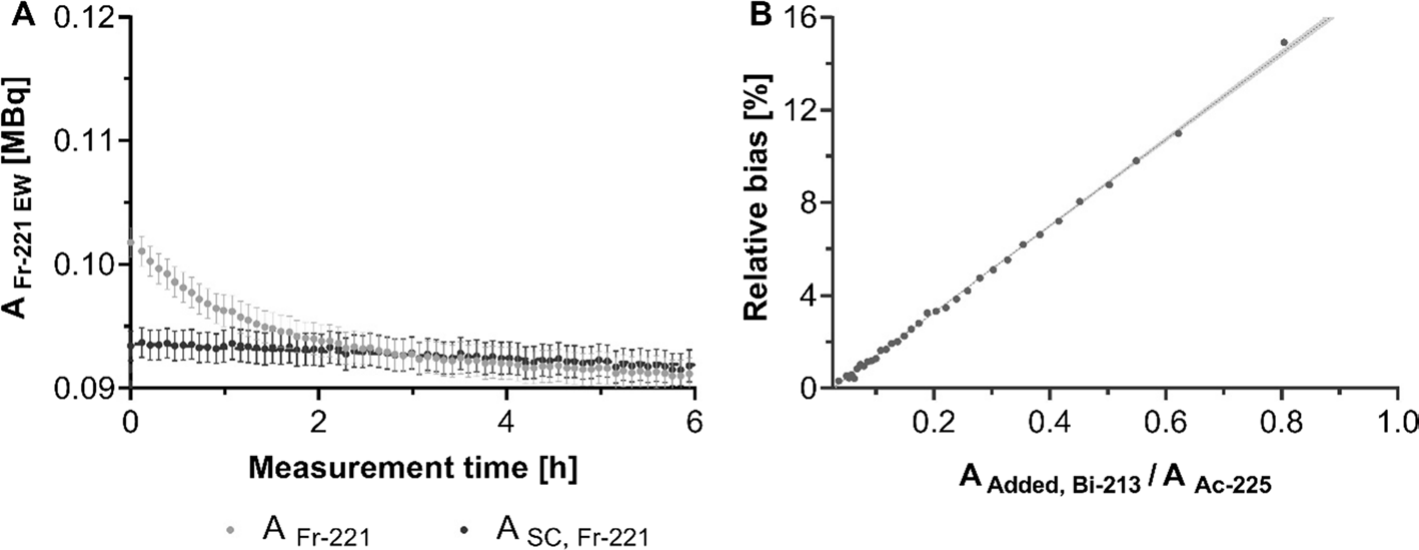
**A** ^225^Ac activities measured with ${\text{EW}}_{{221_{{{\text{Fr}}}} }}$ with and without scatter correction are plotted as function of measurement time for the sample 3 (see Table 1) containing ^225^Ac and additional ^213^Bi activity. Error bars represent the standard deviation provided by the GC software. **B** Relative bias (percentage relative difference) of ^225^Ac activity estimated using ${\text{EW}}_{{221_{{{\text{Fr}}}} }}$ as function of the added ^213^Bi activity (compared to SEq between ^225^Ac and ^213^Bi) when no correction for the additional down scatter of ^213^Bi gamma-emissions in the ${\text{EW}}_{{221_{{{\text{Fr}}}} }}$ is applied

For the single time-point approach, ^225^Ac activities measured with ${\text{EW}}_{{221_{{{\text{Fr}}}} }}$ with and without scatter correction are plotted as function of time for the sample 3 containing ^225^Ac in SEq with ^213^Bi and additional ^213^Bi activity (Fig. 6A). This measurement was started after SEq was reached between ^221^Fr and ^225^Ac (~ 30 min) such that ^225^Ac activity could be estimated from the CPMs measured within ${\text{EW}}_{{221_{{{\text{Fr}}}} }}$. When no scatter correction was applied, ^225^Ac activities showed an overestimation due to the additional down scatter of gamma-emissions by the added ^213^Bi activity. The overestimation gradually decreased because of the decay of this additional ^213^Bi till SEq was again reached between ^225^Ac and ^213^Bi (Fig. 6B). Applying a correction for down scatter of ^213^Bi gamma emissions in the ${\text{EW}}_{{221_{{{\text{Fr}}}} }}$ reduces this overestimation of ^225^Ac activities. For a single time-point GC measurement at 30 min, omission of the scatter correction resulted in an overestimation of 6 to 9% of the ^225^Ac activity while the estimated activity was within 3% of the reference value when scatter correction was applied. When using the estimated ^225^Ac activity to determine the additional ^213^Bi activity, this resulted in an underestimation of 11 to 15% when no scatter correction was applied and while it was within 3% of the reference activity after applying a correction for down scatter (Table 2).

### Quantification of ^225^Ac and ^213^Bi during radiopharmaceutical QC of [^225^Ac]Ac-DEPA and [^225^Ac]Ac-DOTA complexes

An overview of the bound fraction of [^225^Ac]Ac-DEPA/DOTA after a 15 min and 24 h incubation in human serum is given in Table 3. These estimates are based on single time-point GC measurement protocols.

**Table 3:** Bound fraction of ^225^Ac (activity upper part iTLC/activity iTLC) to [^225^Ac]Ac-DEPA/DOTA complexes for samples 5 and 6 (see Table 1) after 15 min and 24 h incubation in human serum

EW	CPM	[^225^Ac]Ac-DOTA		[^225^Ac]Ac-DEPA	
		15 min	24 h	15 min	24 h
${\text{EW}}_{{213_{{{\text{Bi}}}} }}$(at 5 h)	$CPM_{{{\text{ EW}}_{{213_{{{\text{Bi}}}} }} }}$	88%	91%	96%	96%
${\text{EW}}_{{221_{{{\text{Fr}}}} }}$(at 30 min, without scatter correction)	$CPM_{{{\text{ EW}}_{{221_{{{\text{Fr}}}} }} }}$	84%	78%	88%	83%
${\text{EW}}_{{221_{{{\text{Fr}}}} }}$, ${\text{EW}}_{{213_{{{\text{Bi}}}} }}$(at 30 min, with scatter correction)	${\text{CPM}}_{{{\text{ SC,EW}}_{{221_{{{\text{Fr}}}} }} }}$, $CPM_{{{\text{ EW}}_{{213_{{{\text{Bi}}}} }} }}$,	89%	90%	95%	93%

^225^Ac activities were determined with a single time-point GC measurement using ${\text{EW}}_{{213_{{{\text{Bi}}}} }}$ at 5 h, to ensure SEq between ^213^Bi and ^225^Ac, and using ${\text{EW}}_{{221_{{{\text{Fr}}}} }}$ with and without scatter correction at 30 min, to ensure SEq between ^221^Fr and ^225^Ac

**Fig. 7:**
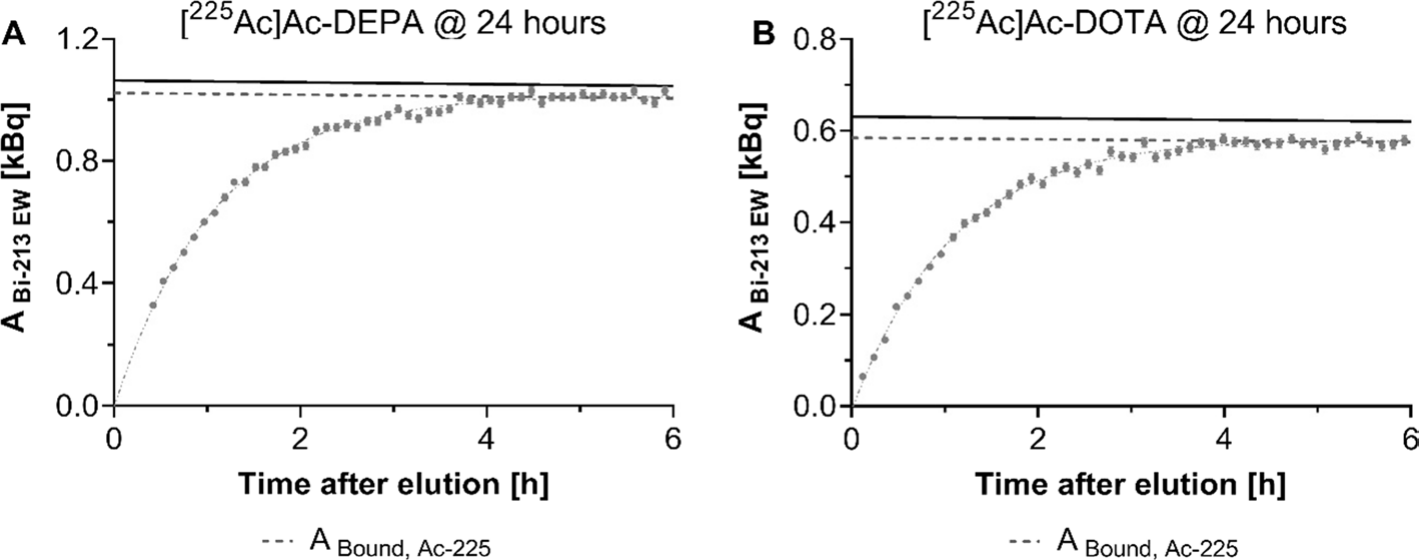
Multiple time-point GC measurements were performed of the upper part of iTLC strip using ${\text{EW}}_{{213_{{{\text{Bi}}}} }}$ before SEq was reached between ^213^Bi and ^225^Ac (< 5 h), to determine the amount of bound ^213^Bi after 24 h of incubation in human serum of [^225^Ac]Ac-DEPA (**A**) and [^225^Ac]Ac-DOTA (**B**). The solid line represents the total amount of ^225^Ac (from GC of both parts of the iTLC strips) and the dotted line the amount of bound ^225^Ac. Error bars represent the standard deviation provided by the GC software

Multiple time-point GC measurements, performed of the upper part of iTLC strip for the 24 h incubation time in human serum using ${\text{EW}}_{{213_{{{\text{Bi}}}} }}$ (see Fig. 7), showed an ingrowth of ^213^Bi for both [^225^Ac]Ac-DOTA and [^225^Ac]Ac-DEPA until SEq was again restored between ^225^Ac and ^213^Bi after 5 h (i.e. ^213^Bi reached ~ 99% of ^225^Ac activity). These findings correspond to a lower fraction of bound ^213^Bi compared to the fraction of bound ^225^Ac.

For [^225^Ac]Ac-DOTA, fitting of the non-linear least squares fitting of a double exponential (i.e. Eq. 6) function to the multiple GC measurements at different time-points estimated the initial ^213^Bi activity in the upper part of the iTLC strip as zero, within a 95% confidence interval (0.01 ± 0.02 kBq), meaning that no bound ^213^Bi was present in these samples after 24 h of incubation in human serum.

## Discussion

This study focused on GC measurements to quantify ^225^Ac activity when SEq is not guaranteed or not yet reached between ^225^Ac and its daughter radionuclide ^213^Bi. Before performing our experiments, we first determined the linear range and calibration factor of the GC system and ensured that all activities for this study were within this range to guarantee optimal quantitative performance. In addition, samples were prepared to have an equal volume of 0.5 mL to avoid a volume effect on the detection efficiency. As GC measurements of ^225^Ac-activity rely on the gamma emissions by daughter radionuclides ^213^Bi and ^221^Fr, this indirect approach requires SEq between ^225^Ac on the one hand and ^213^Bi and/or ^221^Fr on the other hand, especially if only one time-point is measured. When using an EW for ^213^Bi, one should wait for more than 5 h to ensure that ^213^Bi and ^225^Ac activities are in agreement (~ 99%) and bias on ^225^Ac activity measurements is avoided as much as possible. While this long waiting time can prove to be challenging from a practical point of view, it also prevents the quantification of ^213^Bi activities before SEq with ^225^Ac. This information is however needed to monitor the relocation of free ^213^Bi, released from the molecular vector, from the tumor site or tissues showing target expression to organs involved in excretory pathway of ^213^Bi. A straightforward approach to avoid the need for SEq between ^225^Ac and ^213^Bi when using the EW of ^213^Bi for ^225^Ac quantification, is a multiple GC measurement protocol where activities are measured at different time points before and/or during SEq between ^225^Ac and ^213^Bi. As the approach does not require SEq, neither between ^225^Ac and ^213^Bi nor between ^225^Ac and ^221^Fr, GC measurements can be initiated as soon as samples are available to ensure that the highest activity of ^213^Bi can be measured. This can be very useful for biodistribution studies where the ^213^Bi activity in the different organs is not known in advance and sensitivity to pick up even low levels of ^213^Bi activities should be maximized. For our experiments, we performed measurements every 5 min to demonstrate feasibility but in practice, the optimal timing for the measurements will depend on the number of samples which need to be counted and the availability of the GC system. However, we would advise to maximize the number of measurements to reduce the impact of noise, since ^213^Bi activities are expected to be rather low in preclinical biodistribution and radiotoxicity studies on rodents (Kruijff et al. 2019; Miederer et al. 2004a, 2008; Poty et al. 2019; Nedrow et al. 2017; Schwartz et al. 2011). In addition, this approach using multiple GC measurements could also be considered for monitoring whether ^221^Fr activities are different from the activities expected for SEq with ^225^Ac. However, non-SEq between ^221^Fr and ^225^Ac is generally considered irrelevant and challenging to assess, due to its relatively short physical half-life of only a few minutes. Moreover, because ^221^Fr is the first gamma-emitting daughter in the decay chain of ^225^Ac, with this short half-life, it is generally considered as the closest proxy daughter to determine the presence/location and activity of ^225^Ac.

Therefore, we suggested a single GC measurement to determine the ^225^Ac activity using the EW of ^221^Fr. This can be done once SEq is established between ^225^Ac and ^221^Fr around 30 min after mixed ^225^Ac/^213^Bi samples have been synthetized as was previously reported (Hooijman et al. 2021; Pretze et al. 2021). However, we noticed that ^225^Ac activity estimated with the EW of ^221^Fr is biased when the activity of ^213^Bi does not correspond with the expected activity in case of SEq with ^225^Ac. This problem can be addressed by using a HPGe detector due to its superior energy resolution which provides a more definite isotopic identification for low-energy emitters than NaI(Tl) detectors (Perez-Andujar and Pibida 2004). However, the purpose of this study was to optimize the GC protocols because these detectors are much more accessible than HPGe detectors. Moreover, commercial GC systems allow automatic measurements of many samples, making it a very suitable technique for the multiple time point protocols that were used for this study. Therefore, we advise to estimate the down scatter of the higher-energy gamma rays by ^213^Bi into the EW of ^221^Fr when using GC protocols. This way, the count rate in the EW of ^221^Fr can be corrected, such that ^225^Ac-estimates using the EW of ^221^Fr are unbiased and independent of the ^213^Bi activity present in the sample or mixture. Once ^225^Ac activity has been determined, the remaining ^213^Bi activity can be estimated using the EW of ^213^Bi. However, this single GC measurement approach requires a waiting time of around 30 min which reduces the sensitivity for measuring low amounts of ^213^Bi compared to a GC measurement protocol using multiple time points. On the other hand, a single GC measurement protocol can be considered when higher levels of ^213^Bi activities are anticipated, like for example during the synthesis of ^225^Ac-labeled radiopharmaceuticals, or when standardized QC procedures need to be balanced between unbiased activity estimates and short measurement times. A single GC measurement at 2 h after radiolabeling was already suggested as ideal time point for GC measurements to balance the need for a fast release and accurate assessment of the radiochemical yield (Kelly et al. 2021; Eryilmaz and Kilbas 2021). However, one could argue that 2 h waiting time will delay administration to patients, while the bound fraction of both ^213^Bi and ^225^Ac will be reduced to the RDE. Therefore, a GC measurement protocol at 30 min to indirectly quantify ^225^Ac activity by measuring ^221^Fr once SEq is reached between ^225^Ac and ^221^Fr, can be a valid alternative, provided that a correction is applied for the down scatter of the higher-energy gamma emissions by ^213^Bi.

For our study, we used both single and multiple time-point GC measurements to evaluate the stability of [^225^Ac]Ac-DEPA and [^225^Ac]Ac-DOTA constructs in human serum to simulate an in vivo situation and challenge the radiocomplexes to test their stability (e.g. tranchelation by metalloproteins). After 24 h of incubation in human serum, negligible amount of bound ^213^Bi were observed for both constructs based on the GC measurements of the upper part of the iTLC strips just after elution (see Fig. 7). Following the GC measurements, ^213^Bi activity gradually recovered till SEq was reached with ^225^Ac, and ^213^Bi activity was again representative for the fraction of bound ^225^Ac. These findings showed that, after 24 h incubation in human serum, ^213^Bi was not bound to the chelator anymore and was most likely released due to the RDE, while the fraction of bound ^225^Ac remained very high (Kratochwil et al. 2016) and very stable for both constructs (see Table 3). To reduce potential radiotoxicity caused by the RDE, diethylenetriamine pentaacetic acid (DTPA, hydrophilic chelate) can be added to the final formulation buffer for complexation of free ^225^Ac and recoiled daughters before administration and combined with diuretic drugs to increase renal excretion (Kratochwil et al. 2016). However, once administered, the ^225^Ac-labeled compound is being added to a diluted medium, such as blood pool, and ^213^Bi will still be released and cause additional radiotoxicity because of RDE. This can justify, to some extent, the high uptake of free ^213^Bi in healthy (i.e. non-targeted) organs (e.g. kidneys Kruijff et al. 2019; Song et al. 2009; Drecoll et al. 2009) early after injection. Ideally, this risk of potential redistribution and additional radiotoxicity should be minimized by using more stable targeting systems with high radiolabeling yields capable of retaining part of the daughter nuclides or strategies to retain radionuclides in the tumor cells/tissues (Kruijff et al. 2019; Robertson et al. 2018).

In terms of clinical translation, one could consider using the proposed GC protocols to quantify activity levels of ^225^Ac and ^213^Bi in blood and urine samples of patients undergoing ^225^Ac -TAT. This way, renal, bone marrow, urinary bladder wall, or gastrointestinal toxicity can be estimated and whole-body clearance can be monitored in a more patient-specific manner, contrary to the more empirically dose estimates to OARs based on the retrospective evaluation of radiotoxicity and treatment response in groups of patients (Siegel et al. 1999). Moreover, accurate GC measurements of the ^225^Ac and ^213^Bi activity levels in patient samples could be combined with compartmental models to unravel the biokinetics of ^225^Ac-pharmaceuticals and recoiling daughters for a better dosimetry (Hooijman et al. 2021), such to allow better prediction of radiotoxicity and treatment efficacy in patients.

The main limitation of this study is the limited number of experiments because of the limited availability of ^225^Ac. Therefore, the proposed GC protocols should be further validated and especially the time schedule of the multiple GC measurements for each specific application.

## Conclusions

For this study, we evaluated two GC measurement protocols for quantifying ^225^Ac-activity when no SEq is guaranteed between ^225^Ac and its daughter radionuclide ^213^Bi. A first protocol performed multiple measurements using only the ^213^Bi energy window and requires no secular equilibrium between ^225^Ac and its gamma emitting daughter radionuclides. For the second protocol, SEq was required between ^225^Ac and ^221^Fr corresponding to a waiting time of around 30 min but only one measurement was needed using both the ^213^Bi and ^221^Fr energy window. Both protocols were able to accurately estimate ^225^Ac-activities provided the ^221^Fr energy window was corrected for the down scatter of the higher-energy gamma-emissions by ^213^Bi. In addition, these two protocols were able to quantify ^213^Bi-activities which cannot be attributed to the parent-daughter decay of ^225^Ac. From this perspective, a multiple GC measurement protocol could prove more sensitive to pick up low levels of ^213^Bi since it doesn’t require SEq and allows measurements as soon as samples are available. This could prove beneficial to study the recoil daughter effect and redistribution of free ^213^Bi by monitoring the accumulation or clearance of ^213^Bi in different tissues during biodistribution studies or in patient samples during clinical studies. On the other hand, the single GC measurement protocol, although required a 30 min waiting time, is more time and cost efficient and therefore more appropriate for standardized QC procedures of ^225^Ac-labeled radiopharmaceuticals.

## Supplementary Information

**Additional file 1: Fig. S1.** Relative counting efficiency of the GC as function of sample volume for the quantification of ^225^Ac using the ^213^Bi and ^221^Fr EW. Design and results of an additional experiment to study the effect of sample volume on the relative GC detector efficiency.

## Data Availability

All data generated or analysed during this study are included in this published article and its supplementary information files.

## Abbreviations

CF: Calibration factor
CPM: Counts per minute
DOTA: 1,4,7,10-Tetraazacyclododecane-1,4,7,10-tetracetic acid
DEPA: 7-[2-(Bis-carboxymethyl-amino)-ethyl]-4,10-bis-carboxymethyl-1,4,7,10-tetraazacyclododec-1-yl-acetic acid
DTPA: Diethylenetriamine pentaacetic acid
EW: Energy window
GC: Gamma counter
iTLC: Instant thin-layer liquid chromatography
LET: Linear energy transfer
QC: Quality control
RCY: Radiochemical yield
RDE: Recoil daughter effect
SEq: Secular equilibrium
SPECT: Single photon emission computed tomography
TAC: Time–activity curves
TAT: Targeted alpha-particle therapy
