# Supplementary material for: Gamma counting protocols for the accurate quantification of 225Ac and 213Bi without the need for a secular equilibrium between parent and gamma-emitting daughter
Source: EJNMMI Radiopharm Chem. 2022 Oct 23;7:28. doi: 10.1186/s41181-022-00174-z (PMC9588853; doi:10.1186/s41181-022-00174-z)
Supplement: Supplementary file 1 — Additional file 1: Fig. S1. Relative counting efficiency of the GC as function of sample volume for the quantification of 225Ac using the 213Bi and 221Fr EW. Design and results of an additional experiment to study the effect of sample volume on the relative GC detector efficiency. [file 41181_2022_174_MOESM1_ESM.docx]

**Supplemental material**

The accuracy of GC measurement can be affected by differences in the sample volume. We measured this effect for ^225^Ac using a range of volumes varying from 0.15 – 3.5 mL (in triplicates) and using both the ^213^Bi and ^221^Fr EW (see Supplementary Figure 1). A fixed volume of 0.15 mL corresponding to a ^225^Ac-activity of around 8 kBq was added to all the samples to keep the total activity constant. The activity in each sample was less than 10 kBq at the start of the experiment to ensure linear GC response. To assess the relative efficiency, each value was divided by the measurement corresponding to the sample with smallest volume (0.15 mL).


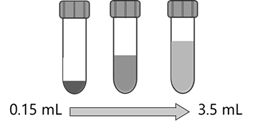

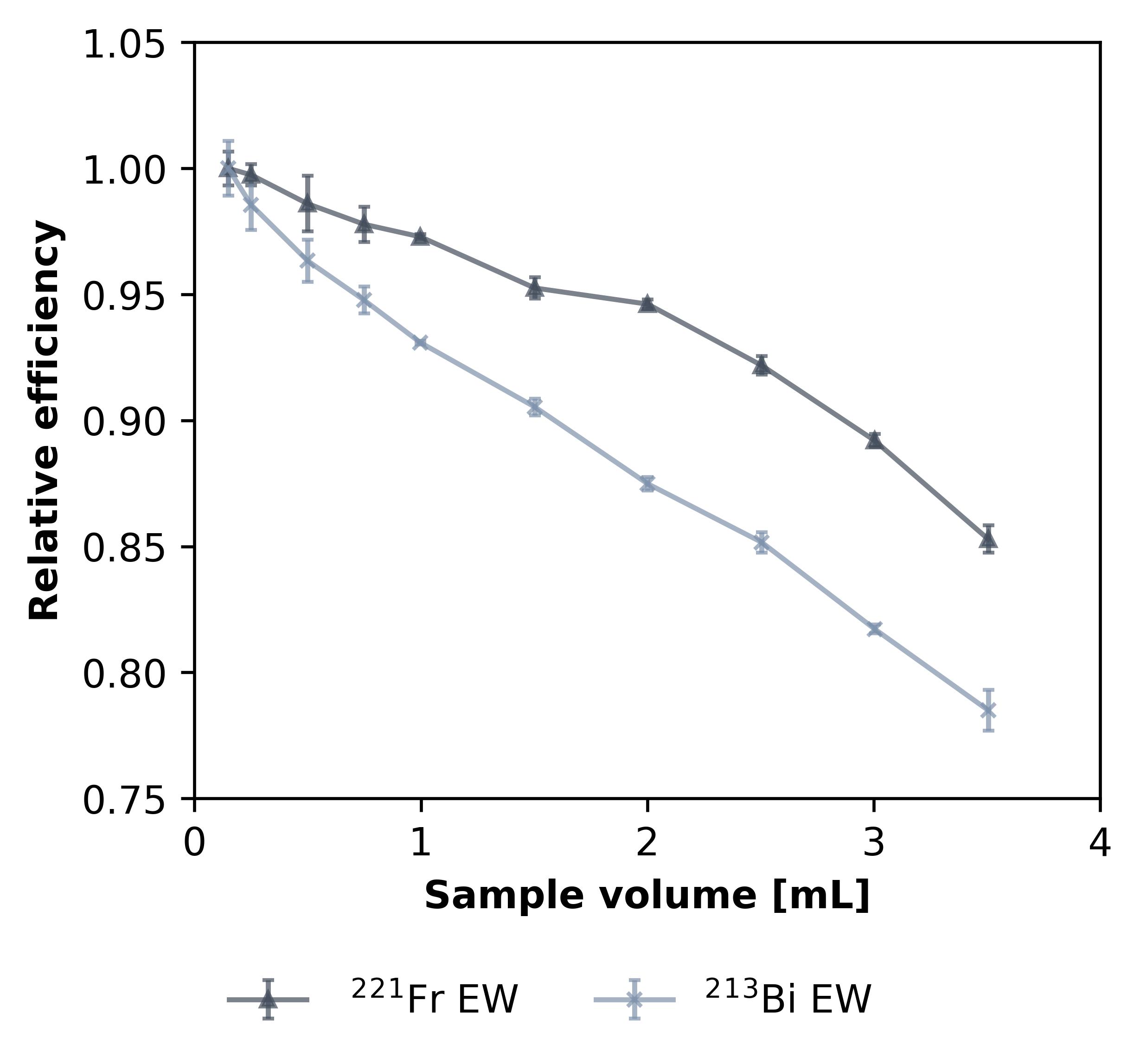


**Supplementary Figure 1:** Relative counting efficiency of the GC as function of sample volume for the quantification of ^225^Ac using the ^213^Bi and ^221^Fr EW. Error bars represent standard errors (n=3).

Results demonstrated that samples with volumes smaller than 1.5 mL will not be considerably affected by a loss of detection efficiency (Relative efficiency > 90%). However, if the sample volumes are expected to be 3 - 4 mL, which could be the case for tissue samples of biodistribution studies or patient blood samples [1], one can expect a considerable loss of detection efficiency up to more than 20% for the ^213^Bi EW for volumes larger than 3 mL. This loss of detection efficiency for larger samples is more pronounced for GC measurements using the ^213^Bi EW compared to the ^221^Fr EW which is in line with current literature data [2].

[1] Mazzaglia S, Stella G, Tonghi LB, Tuvé CN, Politi G, Pellegriti G, et al. Absorbed Dose Evaluation in Radioiodine Therapy with Different Approaches n.d. https://doi.org/10.3390/instruments3030039.

[2] Lodge MA, Holt DP, Kinahan PE, Wong DF, Wahl RL. Performance assessment of a NaI(Tl) gamma counter for PET applications with methods for improved quantitative accuracy and greater standardization. EJNMMI Phys 2015;2:1–13. https://doi.org/10.1186/s40658-015-0114-3.
